# Domestic

**Published:** 1841-11-27

**Authors:** 


					﻿DOMESTIC.
In relation to the condition of distant schools
the intelligence that flows in upon us through
public channels, comes often in “such a ques-
tionable shape, we cannot even speak it.”
When the struggle of competition is over for
the season, the number of matriculations is ac-
curately ascertained. But who shall, even
then, determine the number of men of straw
that are made to assist in swelling the nominal
amount of the class 1 As for the flourishes of
trumpets in newspapers and introductories
about the peculiar advantages of particular
colleges, we estimate them—if at all—in in-
verse proportion to their loudness. The school
or the teacher that swells largest on paper, is
usually the first to explode in practice. From
private sources we derive some facts. The
old school of New York has certainly about
one hundred pupils, and a prospect before it
unusually bright. Of the class of the new
school, we know nothing authentic from disin-
terested testimony, and are not inclined to
guess. The cla.ss of Louisville numbers about
two hundred and fifty. Our own appears a
very little diminished in numbers, from the
absence of the usual number; of established
practitioners who visit us to review and ex-
tend their knowledge. The number of stu-
dents of the first and second course does not
vary appreciably from the average. R. C.
Interments in the City and Liberties of Phi-
ladelphia, from the 13th to the 20th of No-
vember.
>	E	‘	>	o
s'.	b.	2".
Diseases. £ g; Diseases. £ K
“	(T	*	rt>
_____ _______ “ ?
Asthma,	1	Brought forward,38 33
Abscess kin the	Malformation of
breast,	0	1	the Heart,	0	1
Apoplexy	2	o'	Mortification of
Burns,	0	1	the stomach,	1	0
Casualty,	1	0	Mania a Potu,	1	0
Croup,	0	3	Morbus Cole-
Consumption of	reus,	0 1
the lungs,	16	3	Old age,	2	0
Contusion,	1	6	Palsy,	2	0
Dropsy, abdomi-	Scirrhus of Bylo-
nal,	1	0	rus,	1	0
----- Head,	0 1 Smallpox,	2 6
----------------------------------------Breast,	3 0 Still-born,	0 13
Disease of the	Tetanus,	0	1
brain,	0	1 Teething,	0	1
----- Heart,	1 0 Unknown,	0 1
	Lungs, 0	1		 _
--------------------------------------------Bowels,	0 1 Total, 104—47 57
Dysentery,	2	0	_
Debility	0	2	Of the above, there
Fever,	2	0	were under 1 year, 29
----- Brain, 0	1 From 1 to 2	8
	Remittent,	0 2	2	to	5	10
-------------------------------------------Puerperal,---------------------------------10-------------------------------5------------------------------to----------------------------10--------------------------8
-------------------------------------------Scarlet,	0	1	10	to	15	1
Gangrene,	10	15 to 20	1
Inflammation of	20 to 30	12
the Brain,	2 0	30 to 40 19
-----Bronchi, 0	1	40	to	50	5
	Lungs, 1	5	50-----to---60-3
------------------------------------------------------------------------------------------------------------------------Stomach and	60	to	70	1
Bowels,	1	0	70 to 80	1
-----Bowels, 1	0	80 to 90	4
------------------------------------------Peritonaeum,0	1	90 to 100	0
Intemperance, 1	0	100 to 110	2
Marasmus, 0 2	__________________________________
---------------------Total,	104
Carriedforward, 38 331
				

